# Diagnosis, Classification and Management of Mast Cell Activation Syndromes (MCAS) in the Era of Personalized Medicine

**DOI:** 10.3390/ijms21239030

**Published:** 2020-11-27

**Authors:** Peter Valent, Cem Akin, Boguslaw Nedoszytko, Patrizia Bonadonna, Karin Hartmann, Marek Niedoszytko, Knut Brockow, Frank Siebenhaar, Massimo Triggiani, Michel Arock, Jan Romantowski, Aleksandra Górska, Lawrence B. Schwartz, Dean D. Metcalfe

**Affiliations:** 1 Department of Internal Medicine I, Division of Hematology and Hemostaseology and Ludwig Boltzmann Institute for Hematology and Oncology, Medical University of Vienna, 1090 Vienna, Austria; 2 Division of Allergy and Clinical Immunology, University of Michigan, Ann Arbor, MI 48106, USA; cemakin@med.umich.edu; 3 Department of Dermatology, Medical University of Gdansk, 80-211 Gdansk, Poland; 4 Allergy Unit, Verona University Hospital, 37126 Verona, Italy; patrizia.bonadonna@ospedaleuniverona.it; 5 Division of Allergy, University Hospital Basel and University of Basel, 4031 Basel, Switzerland; karin.hartmann@usb.ch; 6 Department of Allergology, Medical University of Gdansk, 80-211 Gdansk, Poland; mnied@gumed.edu.pl (M.N.); jromant@gumed.edu.pl (J.R.); agorska@gumed.edu.pl (A.G.); 7 Department of Dermatology and Allergy Biederstein, Technical University of Munich, D-80802 Munich, Germany; knut.brockow@tum.de; 8 Dermatological Allergology, Department of Dermatology and Allergy, Charité—Universitätsmedizin Berlin, Corporate Member of Freie Universität Berlin, Humboldt-Universität zu Berlin, and Berlin Institute of Health, 10117 Berlin, Germany; frank.siebenhaar@charite.de; 9 Division of Allergy and Clinical Immunology, University of Salerno, 84131 Salerno, Italy; mtriggiani@unisa.it; 10 Department of Hematological Biology, Pitié-Salpêtrière Hospital, Pierre et Marie Curie University (UPMC), 75005 Paris, France; michel.arock@aphp.fr; 11 Department of Internal Medicine, Division of Rheumatology, Allergy & Immunology, Virginia Commonwealth University, Richmond, VA 23284, USA; lschwart@vcu.edu; 12 Laboratory of Allergic Diseases, NIAID, NIH, Bethesda, MD 20852, USA; dmetcalfe@niaid.nih.gov

**Keywords:** Mast cell activation syndrome, Hereditary alpha tryptasemia, Mastocytosis, IgE

## Abstract

Mast cell activation (MCA) is seen in a variety of clinical contexts and pathologies, including IgE-dependent allergic inflammation, other immunologic and inflammatory reactions, primary mast cell (MC) disorders, and hereditary alpha tryptasemia (HAT). MCA-related symptoms range from mild to severe to life-threatening. The severity of MCA-related symptoms depends on a number of factors, including genetic predisposition, the number and releasability of MCs, organs affected, and the type and consequences of comorbid conditions. In severe systemic reactions, MCA is demonstrable by a substantial increase of basal serum tryptase levels above the individual’s baseline. When, in addition, the symptoms are recurrent, involve more than one organ system, and are responsive to therapy with MC-stabilizing or mediator-targeting drugs, the consensus criteria for the diagnosis of MCA syndrome (MCAS) are met. Based on the etiology of MCA, patients can further be classified as having i) primary MCAS where *KIT*-mutated, clonal, MCs are detected; ii) secondary MCAS where an underlying IgE-dependent allergy or other reactive MCA-triggering pathology is found; or iii) idiopathic MCAS, where neither a triggering reactive state nor *KIT*-mutated MCs are identified. Most severe MCA events occur in combined forms of MCAS, where *KIT*-mutated MCs, IgE-dependent allergies and sometimes HAT are detected. These patients may suffer from life-threatening anaphylaxis and are candidates for combined treatment with various types of drugs, including IgE-blocking antibodies, anti-mediator-type drugs and MC-targeting therapy. In conclusion, detailed knowledge about the etiology, underlying pathologies and co-morbidities is important to establish the diagnosis and develop an optimal management plan for MCAS, following the principles of personalized medicine.

## 1. Introduction

Mast cells (MCs) are tissue-fixed effector cells involved in the initiation and perpetuation of allergic inflammation, as well as in a number of other inflammatory states [1,2,3,4,5]. In common with blood basophils, MCs express high-affinity IgE-binding sites and produce both pro-inflammatory and vasoactive mediators, some of which are stored in their metachromatic secretory granules (Table 1) [1,2,3,4,5]. During a severe anaphylactic reaction, allergen-induced cross-linking of IgE-binding sites (FceRI) on MCs is followed by an explosive release of granular-associated mediators. In addition, activated MCs release newly formed cell membrane-derived (lipid-type) mediators of hypersensitivity reactions and cytokines into the extracellular space [1,2,3,4,5]. Blood basophils may also participate in allergic and other inflammatory reactions in the same way as MCs [1,3,6]. However, not all hypersensitivity reactions may involve both cell types, even if the reaction is systemic. In addition, some of the mediators involved in anaphylactic reactions are produced and released primarily by MCs, but not by basophils. 

The ability of MCs and basophils to liberate mediators of anaphylaxis in the context of cell activation, also known as ‘releasability’, depends on several different factors, including the underlying (primary) disease/pathology, the numbers and type of activated surface receptors and signaling molecules, and the genetic pattern [7,8,9,10,11]. The severity of a hypersensitivity reaction is dependent on additional variables, such as the number of MCs (and sometimes also basophils) involved in the reaction; presence and type of triggering allergen; the amount and type of allergen-specific IgE; triggering cofactors, like exercise, acetylsalicylic acid or alcohol; presence of comorbidities; the local (tissue) microenvironment; and the cytokine and chemokine networks involved [1,2,3,4,12,13,14,15,16,17].

MC activation (MCA) occurs in a number of pathologic conditions. Acute MCA is commonly seen in allergic reactions and may be associated with clinical signs and symptoms of anaphylaxis [1,2,3,4,5,12,13,14,15,16,17]. Severe or even life-threatening MCA may occur when the burden of MCs is high or/and these cells are in a ‘hyperactivated’ state [15,16,17]. In such patients, a MCA syndrome (MCAS) may be diagnosed when the symptoms are recurrent and MCAS criteria are fulfilled [15,16,17,18,19,20,21,22,23]. Historically, clinical symptoms arising from MCA have primarily been studied in the context of allergic/atopic disorders [1,2,3,4,5]. More recently, however, MCA has also been studied in the context of other pathologic conditions, including systemic mastocytosis (SM) [2,4,5,12,13,14,15,16,17,18,19,20,21,22,23].

During the past 10 years, criteria for MCA and MCAS have been developed by an international (EU/US-based) consensus group consisting of experts in the fields of allergy, dermatology, hematology, pathology, and molecular medicine [18,19,20,21,22]. These MCAS criteria have been validated in various studies [24,25,26]. In addition, diagnostic algorithms and recommendations for clinical management and therapy of patients with MCAS have been published recently [23].

In the current article, these criteria and the classification of MCAS as well as management strategies are discussed in light of new developments in the field; the growing number of available clinical, serological and biochemical markers; and the emerging medical need to diagnose and manage these patients based on scientific evidence and according to the principles of personalized medicine.

## 2. Symptoms recorded in Patients with MCA

MCA-related symptoms range from mild headache or urticaria to severe or even life-threatening anaphylaxis. These symptoms are caused by a number of vasoactive and pro-inflammatory mediators released from MCs (or alternatively basophils) when these cells are activated by an allergen via IgE and IgE receptors, or by other triggers [1,2,3,4,5,12,13,14,15,16,17]. An overview of MC-derived mediators and their clinical impact are shown in Table 1.

As mentioned, the severity of MCA-related symptoms depends on a number of independent factors and correlates with the amount and type of mediators released from MCs during an MCA event. In general, the symptoms that can be provoked by MC mediators are manifold and the type of MCA can be divided into (i) local versus systemic, (ii) acute versus chronic, and (iii) mild versus severe (Table 2) [23]. 

Typical manifestations of immediate-type hypersensitivity (allergic) reactions consistent with the diagnosis of systemic MCA (anaphylaxis), include acute urticaria, flushing, pruritus, headache, abdominal cramping, diarrhea, respiratory symptoms, and vascular instability (hypotension) [20,21,22,23]. Although none of these symptoms are absolutely specific for MCA, many are typically found in these patients. Especially, when occurring together in one patient at the same time, these symptoms are suggestive of MCA, although basophil activation is considered to manifest with a similar spectrum of symptoms. The likelihood of MCA is even higher when multiple (two or more) of these symptoms are recorded and are responsive to drugs blocking MC mediator effects, mediator production, or mediator release in MCs. Responsiveness to such intervention has been proposed as a diagnostic criterion of MCAS [20,21,22]. This is particularly relevant when the symptoms are unusual or when the patient has another unrelated disease that may cause symptoms mimicking (or resembling) MCA. Another important consideration is that several different mediators may be involved in MCA-related symptoms [1,2,3,4,5,17,18,19,20,21,22]. Likewise, severe hypotension may be triggered by both histamine and prostaglandin D2 (PGD_2_) derived from activated MCs in the same patient (Table 1).

Whether MCAS may also present as a chronic disease in the absence of recognized systemic anaphylactic events is controversial. Non-specific symptoms such as headache, fatigue, nausea and sleep disturbance do not fulfill well-established criteria of MCA and therefore of MCAS. Nevertheless, these symptoms are clinically relevant and thus should be treated appropriately to provide symptomatic relief. Also, it is important to recognize that the differential diagnoses to be considered in such patients are broad, including neurologic, infectious and cardiac disorders [20,21,22,23].

Finally, MCA may occur as a local, non-systemic event, which may also represent a clinical challenge for the treating physician. MCAS criteria are not fulfilled in these patients (Table 2). Examples include MCA in the skin presenting as urticaria or MCA in the lungs presenting as bronchial asthma. Although the MCAS criteria are not fulfilled, the impact of local MCA in these conditions needs to be acknowledged, and specific therapy using anti-mediator type drugs or MC-stabilizing agents may be required. On the other hand, it often remains unclear whether MC are indeed involved and if yes, to what extent. In addition, the etiology of local MCA often remains unknown. Whether local MCA is triggered by various infectious or toxic reactions is certainly worth considering, but precise criteria are needed to prove such an association and will be important to develop. Such criteria could also lead to better therapeutic interventions.

## 3. Laboratory Assessments in Patients with Systemic MCA

MCA is typically accompanied by the release of preformed and newly produced vasoactive and pro-inflammatory mediators and other MC-dependent compounds [1,2,3,4,5]. In most patients with severe systemic reactions, increased levels of MC-derived mediators are measurable in biological fluids (serum, plasma, urine) [20,21,22,23,24,25,26]. Some of these mediators, such as tryptase or prostaglandin D2, are more specific for MCs [1,2,3,4]. Other mediators are less specific and also produced by other cell types. Whereas histamine is released from MCs and basophils in similar quantities, tryptase is considered rather specific for MCs, although basophils can also express and release small quantities of the enzyme [27,28,29,30]. Therefore, most experts agree that for daily clinical practice, a rapid increase in the serum tryptase level from the individual’s baseline is MCA-specific and thus a reliable diagnostic parameter [20,21,22,23,24,25,26,31,32]. If no pre-therapeutic baseline is available, the baseline serum tryptase level has to be assessed after complete recovery (at least 24–48 h after complete resolution of symptoms) or in a symptom-free interval [20,21,22,23].

Apart from tryptase, other mediators, when increasing transiently over baseline during a clinically defined attack (MCA-related event), may also serve as supportive evidence of systemic MCA. These include, among others, histamine (plasma, urine), histamine metabolites (urine) and prostaglandin D2 metabolites (Table 3) [31,32,33,34,35,36,37]. 

However, these mediators as noted are less specific for MCA when compared to tryptase. Moreover, no threshold criteria have been proposed and validated to define what minimal increase of these mediators would count as a reliable indicator of systemic MCA (Table 3). Nevertheless, some of these mediators, such as histamine metabolites or prostaglandin D_2_ metabolites, may be helpful in the evaluation and ultimate diagnosis of MCA and should therefore be considered, especially when the serum tryptase assay is not available or when results are equivocal (Table 3). Other mediators are under preclinical or clinical evaluation. One of these mediators is diamine oxidase (DAO) (Table 3) [38]. Indeed, DAO levels and tryptase levels appear to increase in parallel during an MCA event [38]. It does remain unclear, however, whether DAO is indeed produced by MCs or by other cell types involved in these patients. Alternative mediators that have been discussed in the past (or more recently) may not qualify as robust indicators of MCA (Table 3).

## 4. Biochemical Indication of Substantial Systemic MCA 

The normal (physiologic) serum tryptase level determined in healthy adults ranges between 0 and 11.4 ng/mL. Note that tryptase genotyping should be considered in any individual with a tryptase level ≥8 ng/mL to evaluate for HAT which, if present, will affect baseline tryptase levels and is further discussed below under “Impact of Genetic Predisposition in MCA and MCAS”. During a systemic MCA event that involves multiple organ systems and leads to the clinical symptoms of anaphylaxis, a substantial, event-related increase in tryptase over the individual’s baseline is typically found [20,21,22,23,31,32]. Such an increase in tryptase is measurable in patients with MCAS regardless of the underlying etiology or serum basal tryptase (sBT) level. Therefore, a diagnostic increase in tryptase is the most important criterion of MCAS. Because of the different levels in basal tryptase in these patients, a generally applicable equation has been developed by the consensus group [21,22,23]. By this consensus, a minimal increase of tryptase to plus 20% of baseline plus absolute 2 ng/mL (=sBT × 1.2 + 2), meets the definition of a substantial systemic MCA and is thus regarded as biochemical criterion of MCAS [21,22,23]. For example: the baseline tryptase level (sBT) is 10 ng/mL: an increase to 20 ng/mL is suggestive of MCA [1.2 × 10 + 2(absolute) = 14 ng/mL)], qualifying this as a MCA event (biochemical evidence of MCAS). The consensus equation works in all patient groups regardless of the baseline serum tryptase level (normal, slightly elevated or high), has been validated in several studies, and is regarded an acceptable standard in the diagnosis of MCAS (Table 3) [20,21,22,23,24,25,26].

As mentioned before, an increase in other MC-related biochemical parameters may also indicate systemic MCA. For example, a substantial increase in histamine metabolites or prostaglandin D2 metabolites should be regarded as indication of MCA [34,35,36,37]. Currently, a discussion is ongoing to define the minimal diagnostic threshold levels of these markers to qualify as MCAS criterion.

## 5. Diagnostic Consensus Criteria of MCAS 

**Figure 1:**
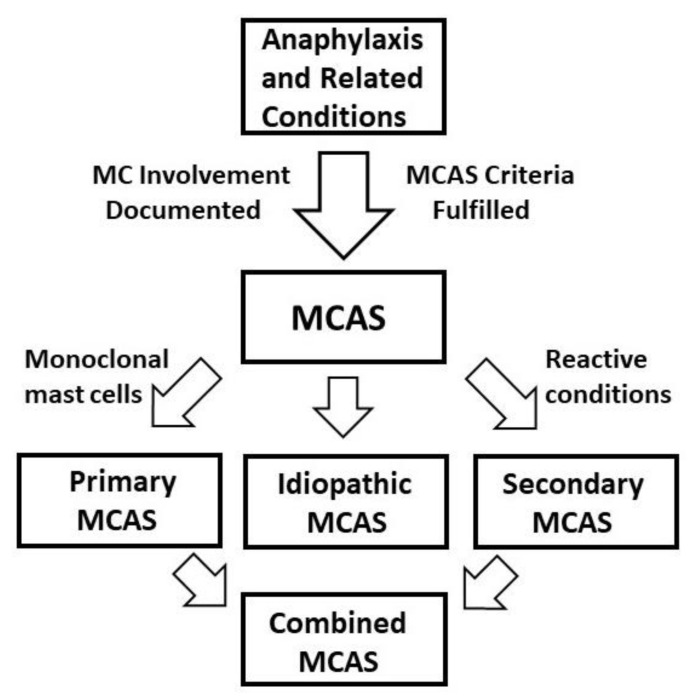
Algorithm in Patients with Suspected MCAS.

Minimal diagnostic criteria of MCAS include (i) typical clinical symptoms indicative of multi-organ involvement, usually meeting criteria for anaphylaxis; (ii) an increase in the serum tryptase level above the individual’s baseline serum tryptase (sBT), meeting the 20% + 2 threshold (= sBT × 1.2 + 2) and/or a clear increase in another MC mediator in biological fluids; and (iii) response of the symptoms to drugs targeting MC activation, mediator release from MCs and/or MC mediator effects (Supplemental Table S1). All three criteria must be fulfilled to arrive at the diagnosis MCAS by consensus criteria [20,21,22,23,39]. Prior to the diagnosis of MCAS, the physician may have established the initial diagnosis as ‘anaphylaxis’. However, a solid confirmation of the involvement and impact of the MC lineage is needed to establish the final diagnosis of MCAS (Figure 1).

In a first step, it is important to document mast cell (MC) lineage involvement and to confirm that MCAS criteria are fulfilled. The most important biomarker is tryptase, a portion of which is stored in secretory granules of MCs. During severe systemic MC activation (MCA), tryptase increases in biological fluids, including serum or plasma. When the diagnosis MCAS is established, it is important to ask whether the patient is suffering from a known primary disorder affecting MCs (clonality due to a somatic *KIT* gain-of-function mutation), secondary MCAS whereby MCs manifest an excessive response to an IgE-dependent allergen or to other IgE-independent agonists, and idiopathic MCAS where no underlying etiology can be documented. In some patients with mastocytosis, a combined form of MCAS (= primary and secondary MCAS) is diagnosed. These patients are at a very high risk to develop severe, life-threatening anaphylaxis. Abbreviations: MCAS, mast cell activation syndrome; MCs, mast cells.

When there is clear indication of MC involvement but the formal criteria of MCAS are not met, the condition may be considered to show MCA or even a (mild or chronic) MC activation disorder. However, such ‘sub-MCAS’ conditions are difficult to diagnose and difficult to separate from unrelated conditions in the differential diagnoses, as no criteria for these pathologies exist. The term ‘syndrome’ and thus MCAS should be reserved for patients who demonstrably suffer from significant MCA-related events meeting MCAS criteria.

There are also conditions that predispose individuals to MCA events and thus to MCAS, including hereditary alpha tryptasemia (HAT) and/or systemic mastocytosis (SM), which also can result in more severe IgE-dependent reactions, particularly to insect venoms (Supplemental Table S2) [21,22,23,40,41,42,43,44,45,46,47,48]. In SM, these patients may be labeled with the appendix SY (symptomatic) when they require continuous interventional treatment with MC stabilizers or mediator-targeting drugs, even if no MCAS is diagnosed [21]. For example, a patient with indolent SM (ISM) and repeated anaphylaxis after hymenoptera stings (but with only a slight increase in tryptase levels) should be labeled as ISM_SY_ although MCAS criteria are not met.

## 6. Differential Diagnoses and Robustness of the Consensus Criteria

A number of medical conditions and disorders can mimic MCAS clinically [20,21,22,23]. These include, among others, infectious diseases, autoimmune disease, cardiac disorders, endocrinologic diseases, neurologic diseases, psychiatric disorders, and intoxications (Table 4).

The key problem is that the symptoms and symptom-patterns in MCAS and in these conditions are often overlapping and that it is often difficult to confirm MC involvement, especially when no MC-specific biochemical markers are available. Table 4 shows a compilation of specific medical conditions that may be confused with MCAS based on overlapping symptoms when only clinical criteria were applied. Therefore, it is of great importance to apply robust, MCA-indicative criteria and to base the diagnosis MCAS on solid diagnostic assays.

A particular problem is that the serum tryptase test is not always available during or after a systemic attack. In these cases, the diagnosis MCAS cannot be established, but the patients may be diagnosed as having had an episode of anaphylaxis, and only subsequent studies may or may not lead to the final diagnosis of MCAS (Figure 1).

Another specific problem is the co-incidence of MCAS with other underlying conditions that could mimic MCAS, for example a severe cardiac disease where hypotension and syncope have been recorded (e.g., aortic valve stenosis plus MCAS). Based on this (hypothetical) possibility, the criteria of MCAS should not include an ‘exclusive’ element. Rather, such patients have to be managed by an interdisciplinary approach, and only a close collaboration with specialists such as cardiologists and experts in MCAS/allergy will be able to establish an optimal treatment plan.

All in all, we opine that the optimal approach is i) to base MC involvement on a solid biochemical marker like tryptase and ii) to establish the diagnosis MCAS only in those patients in whom a substantial (event-related) increase in tryptase over the individual’s baseline (20% + 2 formula = 1.2 * sBT+2 = 1.2 × sBT + 2) has been documented [20,21,22,23]. This approach is most likely to confirm MCAS with high specificity, though the sensitivity will vary, depending on factors such as the clinical severity of the reaction, particularly with respect to hypotension, and the timing of acute sample collection, as sensitivity declines the longer is the time of collection after the peak, which usually occurs about 1 h after onset of clinical signs or symptoms, and post-peak levels decline with a half-life of about 2 h. Other diagnostic models in which only clinical parameters are employed with no robust biochemical makers integrated do not confirm MC involvement and thus are not suitable to differentiate between MCAS and MCAS-mimickers with certainty. This is a critical point, as patients may suffer from other relevant diseases that may need to be diagnosed and treated without delay.

During the preparation of this article, we reviewed other diagnostic criteria and models that have been proposed in the MCAS context in the recent past. In some of these proposals, the authors intertwine diagnostic MCAS criteria with diagnostic criteria of SM. It is worth noting in this regard that neoplastic MCs in SM are often silent clinically (without therapy) so that the patient does not suffer from MCA. In addition, although KIT is activated by certain mutations, MCs in SM have been reported to not exhibit increased releasability compared to normal MCs and the same holds true for ROSA^KITD816V^ cells compared to ROSA^KITWT^ cells [49]. Therefore, we are of the opinion that MC clonality and abnormal MC morphologies or phenotypes do not qualify as MCAS criteria.

## 7. Underlying Disorders and Classification of MCAS

MCAS can be divided into several different variants, depending on the underlying condition and pathology. In particular, patients with MCAS are grouped into primary (clonal = monoclonal) MCAS, secondary MCAS and idiopathic MCAS (Supplemental Table S3) [20,21,22,23]. Primary MCAS is defined by the presence of clonal MCs exhibiting a KIT-activating mutation in the *KIT* gene, usually *KIT* D816V. In most of these patients, an underlying cutaneous mastocytosis (CM) or SM is found. However, in a few of these patients, only the clonal MCs can be detected, whereas the full spectrum of criteria to diagnose SM or CM are not (yet) fulfilled [18,19,20,21,22,23]. In other patients, an underlying IgE-dependent allergy, another hypersensitivity disorder or another inflammatory condition associated with MCA may be detected [20,21,22,23,50]. These patients thus suffer from secondary MCAS. If no clonal MCs and no underlying reactive condition (explaining MCA) are found, the diagnosis is idiopathic MCAS and a search for etiology should continue [20,21,22,23]. Finally, as mentioned before, patients may suffer from more than one form of MCAS, for example when they have an IgE-dependent allergy as well as SM. In these patients, the likelihood of life-threatening anaphylaxis is high and the diagnosis MCAS can often be established in symptomatic cases [50]. These patients are often treated with combinations of drugs targeting MCs, MC mediators, and IgE [50,51].

## 8. Impact of Genetic Predisposition in MCA and MCAS

A number of genetic factors, such as germline mutations, polymorphisms, and gene replications are considered to predispose to more severe hypersensitivity reactions, atopic disorders and MCA. Examples are IL-4 and IL-13 gene polymorphisms leading to an excess production of IgE and thus predisposing to more extensive IgE-dependent allergies; or certain filaggrin gene (*FLG*) mutations predisposing to the development of atopic dermatitis [52,53,54,55,56].

A genetically determined increase in the number of MCs and/or production of MC mediators, such as tryptase, have also been discussed as predisposing factors. Recently, a familial form of hyper-tryptasemia has been described. This condition, termed hereditary alpha tryptasemia (HAT), is an autosomal dominant genetic trait caused by increased germline copies of the *TPSAB1* gene encoding alpha-tryptase where baseline tryptase levels increase in a step-wise fashion with each additional TPSAB1 gene replication [46,47,57,58,59]. Individuals with HAT thus have elevated basal serum tryptase levels. Patients with HAT have been reported to variably present with complaints indicating multi-organ disease and MCA. More recent data suggest that HAT is a valid genetic biomarker that associates with more severe mediator-related symptoms and thus MCA in anaphylaxis, IgE-dependent allergies and mastocytosis [46,47,48,55,56,60,61].

Especially in patients who have HAT as well as a co-existing allergy and/or a co-existing clonal MC disorder (mastocytosis), the risk for development of MCAS may be greater [46,47,48,56,60,61]. Based on these studies, HAT can be regarded as a novel emerging genetic biomarker and modifying risk factor for MCA and MCAS in various patient cohorts, including SM and IgE-dependent allergies. It is also worth noting that HAT is more prevalent in SM than in healthy controls or other myeloid neoplasms [48].

## 9. Management of Patients with MCAS

Management of MCAS is based on both prophylactic and (active) therapeutic approaches. First, it is of considerable importance to advise patients and family members (e.g., parents of children) to avoid any agents or situations that might potentially provoke anaphylactic (allergic) reactions [15,16,17,18,19,20,21,22,23,44,50]. MCAS patients are also advised to take prophylactic anti-mediator therapy (e.g., histamine receptor blocker) for their entire life and to carry two (or more) epinephrine self-injectors after having been informed and instructed how to use these injectors in case of an emergency event. In patients with venom-induced (IgE-dependent) anaphylaxis, and thus secondary MCAS, immunotherapy (IT) is usually recommended, with recognition of potential side effects [17,44,50]. Cytoreductive agents are usually not prescribed for treatment of mediator-related events in MCAS. However, in those with a huge burden of MCs (e.g., patients with SSM or ASM/MCL), cytoreductive therapy might be considered. In fact, the frequency of life-threatening MCAS events may decrease substantially after reducing the mass of neoplastic MCs [50,51]. Aspirin has also been considered previously for therapy of anaphylaxis in patients with SM. However, the doses of aspirin necessary to block MC activation are high and cannot be tolerated by many patients. Other drugs include MC-stabilizing agents and glucocorticosteroids. A new emerging class of MC-targeting drugs are broadly acting tyrosine kinase inhibitors (TKI), such as midostaurin or avapritinib [62,63]. Some of these drugs, like midostaurin, block not only MC proliferation but also IgE-dependent (allergen-induced) activation of MCs [62,64]. Therefore, midostaurin may be a promising agent to suppress MCAS events in patients with primary MCAS and SM. Indeed, midostaurin rapidly improves mediator-induced symptoms and the quality of life in patients with advanced SM [65,66]. Another emerging strategy is to deplete specific IgE (e.g., omalizumab) in patients with secondary MCAS who have an underlying IgE-dependent allergy [67,68]. In patients with severe mixed MCAS (primary+secondary MCAS), multiple specific therapies may be required, following the principles of personalized medicine. For example, in patients with a high mass of neoplastic MC, advanced SM, and severe IgE-dependent allergies, combined therapy with a KIT-targeting drug or cladribine and omalizumab may be required to bring MCAS events under control [51].

## 10. Concluding Remarks and Future Perspectives

MCAS is an unusual MC-induced multi-organ condition characterized by clinical signs and symptoms of anaphylaxis or related pathologies. Because of the multiple mimickers of MCAS and a plethora of circulating proposals, it is important to diagnose MCAS using solid criteria through which MC involvement can be documented. In most cases, an event-related increase of the individual’s serum tryptase level over baseline is sufficient to confirm MC involvement. By contrast, the clinical symptoms of MCA are not specific and may be confused with symptoms of cardiac, infectious, neurologic, endocrinologic, or gastrointestinal disease. Once diagnosed, MCAS is classified into primary forms, secondary MCAS and idiopathic MCAS. Whereas, in some cases, initial therapy has to be applied before a definitive diagnosis of MCAS can be established it is important to be aware of this diagnosis and to establish it in the follow-up. The type of therapy varies in patients with MCAS, depending on the underlying disease, type of MCAS, and response to initial therapy. In severe forms and especially those with mixed MCAS, combination therapies are often recommended, including the use of anti-IgE antibodies, MC-targeted agents, and/or allergen-specific immunotherapies.

## Figures and Tables

**Table 1:** Mast Cell-derived Mediators and Related Clinical Symptoms

Symptoms/Pathology	Mediator(s) Potentially Involved *
**Systemic Symptoms**	
Vascular instability	Histamine, LTC_4_, LTE_4_, PGD_2_, PAF
Edema formation	Histamine, VEGF, LTC_4_, LTE_4_, PAF
Tissue remodeling	Cytokines, Proteases (Tryptase, Chymase)
Bleeding tendency	Heparin, Tissue Type Plasminogen Activator
Fever and cachexia	Tumor Necrosis Factor
Eosinophilia, eosinophil infiltration	Cytokines (GM-CSF, IL-5), Chemokines
Lymphocyte infiltration	Cytokines, Chemokines
Neurologic symptoms, fatigue	Cytokines, Histamine
Headache and nausea	Histamine
**Skin**	
Pruritus	Histamine, Cytokines
Urticaria	Histamine, PAF, PGE_2_, PGD_2_
Angioedema	Histamine, Bradykinin
Flushing	Histamine
**GI-Tract**	
Gastric hypersecretion	Histamine
Peptic ulcer disease	Histamine
Cramping, abdominal pain	Histamine, LTC_4_, PAF
Diarrhea	Histamine
**Respiratory System**	
Nasal congestion, wheezing	Histamine
Bronchoconstriction	Histamine, PGD_2_, LTC_4_, LTD_4_, PAF, Endothelin
Secretion of mucus	Histamine, Proteases, PGD2, LTC_4_
Pulmonary edema	Histamine, LTC_4_, PAF
**Skeletal System**	
Bone remodeling	Proteases, Cytokines
Osteoporosis	Heparin, Proteases

* Whereas some of the mast cell mediators (like histamine or heparin) are produced and stored in metachromatic granules, others (PGs/LTs) are produced and released but are not stored. LT, leukotriene; PG, prostaglandin; GM-CSF, granulocyte/macrophage colony-stimulating factor; VEGF, vascular permeability factor; IL, interleukin, GI-Tract, gastrointestinal tract; PAF, platelet-activating factor.

**Table 2:** Classification of Mast Cell Activation (MCA) and Related Conditions.

**i. According to Organs involved and Severity of Symptoms** Systemic MCA * Mild or moderate systemic MCA (MCAS criteria not fulfilled) Severe systemic MCA = MCAS (MCAS criteria fulfilled) Local MCA (mild/moderate or severe) (MCAS criteria not fulfilled)
**ii. According to the Underlying Condition** Primary (clonal) MCA Cutaneous mastocytosis Systemic mastocytosis (SM) 1-2 minor SM criteria but no SM and no CM detected IgE-dependent allergy (or atopy) Organ-specific variants IgE-independent hypersensitivity reactions Other Conditions Reactive conditions (inflammation) Toxic tissue damage (intoxication) Physical, neurologic and others
**iii. According to Frequency and Symptom-Free Intervals** Acute single event With a known trigger (e.g., allergen) Without a known trigger Episodic recurrent With a known trigger (e.g., allergen) Without a known trigger Chronic persistent With a known trigger (e.g., allergen) Without a known trigger

* Systemic MCA involves two or more organ systems.

**Table 3:** Mast Cell Mediators recommended as Markers of Systemic MCA in Practice.

Recommendation Level	Validated Diagnostic Thresholds (Increase from Baseline) Available
**Recommended as First Line Standard:**	
Tryptase (serum)	yes: plus 20%+2 equation
**Recommended as Alternative or Confirmatory:**	
Histamine metabolites (urinary)	no
Prostaglandin D_2_ metabolites (urinary)	no
**Potentially Useful or Under Development:**	
Histamine (plasma)	no
Diamine oxidase (DAO) *	no
Soluble IgE receptor alpha chain	no
**Not Recommended:**	
Heparin	no
Chymase	no
Chromogranin B	no
Bradykinin	no
Stem cell factor	no
Interleukins	no
Chemokines	no
Basogranulin	no
Platelet activating factor (PAF)	no

* So far it remains unclear whether DAO is expressed by or released from mast cells.

**Table 4:** MCAS Mimickers: Differential Diagnoses to MCAS *.

Disorder/Mimicker	Clinical Findings/Symptoms Presenting as Mimicry
**Cardiovascular Mimickers**	
Myocardial Infarction	Hypotension, Shock, Syncope
Endocarditis/Endomyocarditis	Hypotension, Shock
Aortic Stenosis with Syncope	Syncope
Pulmonary Infarction	Dyspnea, Hypotension
**Endocrinologic Mimickers**	
Acute Hypothyroidism	Hypotension, Shock
Acute Hypoglycemia	Hypotension, Shock
Adrenal Insufficiency	Hypotension, Shock
Hypopituitarism	Hypotension, Shock
**Gastrointestinal Mimickers**	
Acute Inflammatory Bowel Disease	Diarrhea, Pain, Dehydration, Hypotension
VIP-secreting Tumor (VIPoma)	Diarrhea, Dehydration, Hypotension
Active Crohn’s Disease or Colitis Ulcerosa	Diarrhea, Pain, Dehydration, Hypotension
Food Intoxication	Diarrhea, Dehydration, Hypotension
**Infectious Disease Mimickers**	
Severe Bacterial or Viral Infections	Septic Shock
Acute Gastrointestinal Infection	Diarrhea, Cramps, Dehydration, Hypotension
Acute Encephalitis / Meningitis	Headache, Confusion, Fatigue, Hypotension
Acute Parasitic Diseases (e.g., Acute Chagas Disease)	Dehydration, Rash, Hypotension
(e.g., Acute Chagas Disease)	Headache, Dyspnea, Hypotension
**Neurologic/Central Nervous System (CNS) Mimickers**	
Epilepsy	Headache, Confusion, Fatigue, Shock
CNS Tumors	Headache, Confusion, Fatigue, Hypotension
Other CNS Diseases	Headache, Fatigue, Hypotension
Intoxication	Headache, Confusion, Fatigue, Hypotension
Somatoform disorders	Headache, Fatigue, Hypotension
Psychiatric conditions	Headache, Confusion, Fatigue, Hypotension
**Cutaneous Mimickers**	
Hereditary or Acquired Angioedema	Angioedema, Rash, Hypotension
Acute Lupus Erythematosus	Rash, Headache, Fatigue
Acute Toxic Dermatoses	Exanthema, Hypotension
**Hematologic Mimickers: Acute Anemia**	
Acute Gastrointestinal Bleeding	Hypovolemic Shock
Massive Hypermenorrhea	Hypovolemic Shock
**Drug-related Mimickers (Adverse Events after Drug Intake)**	
Drug-induced Hypoglycemia	Fatigue, Loss of Consciousness
Drug-induced Hypotension	Hypotension, Shock
Drug-induced Diarrhea	Diarrhea, Cramps, Dehydration, Hypotension
Drug-Induced CNS Damage	Headache, Fatigue, Hypotension, Confusion

* In most instances, symptoms of acute hypotension are recorded. If additional MCA-mimicking symptoms, such as skin lesions, headache and/or diarrhea are found, it is often difficult to separate the pathology from MCA and thus MCAS. Abbreviations: MCA, mast cell activation; MCAS, MCA syndrome; VIP, vasoactive intestinal peptide.

## Supplementary Materials

The following are available online at https://www.mdpi.com/1422-0067/21/23/9030/s1.
