# Supplementary material for: Diagnosis, Classification and Management of Mast Cell Activation Syndromes (MCAS) in the Era of Personalized Medicine
Source: Int J Mol Sci. 2020 Nov 27;21(23):9030. doi: 10.3390/ijms21239030 (PMC7731385; doi:10.3390/ijms21239030)
Supplement: Supplementary file 1 [file ijms-21-09030-s001.pdf]

Supplementary Materials

Table S1. Consensus criteria for mast cell activation syndrome (MCAS) *.

i) Typical clinical signs of severe, recurrent (episodic) systemic mast cell activation are present (often in form of anaphylaxis) (definition of systemic: involving at least two organ systems)
ii) Involvement of MC is documented by biochemical studies: preferred marker: increase in serum tryptase level from the individual´s baseline to plus 20% plus 2 ng/mL **
iii) Response of symptoms to therapy with MC-stabilizing agents, drugs directed against MC mediator production or drugs blocking mediator release or effects of MC-derived mediators ***

* The consensus criteria for MCAS were first published in (27). All three MCAS criteria (i+ii+iii) must be fulfilled to call a condition MCAS.** Other MC-derived markers of MC activation (histamine and histamine metabolites, PGD2 metabolites) have also been proposed but are less specific compared to tryptase. Abbreviations: MC, mast cells; PGD2, prostaglandin D2. *** Example: histamine receptor blockers.

Table S2. Conditions and Disorders (Co-Morbidities) predisposing to MCA/MCAS.

Predisposing Condition	Estimated Frequency of Anaphylaxis / MCAS (% of patients affected)
**Isolated**:	
Hereditary Alpha Tryptasemia (HAT)	5–10%
Hymenoptera Venom Allergy (HVA)	10–20%
Other IgE-Dependent Allergies (IgE-A)	
Atopic Dermatitis (AD)	10%
Other Atopic Disorders	
Cutaneous Mastocytosis (CM)	<5%
Systemic Mastocytosis (SM)	<5%
**Combined** *:	
HAT + CM	10%
HAT + SM	10%
IgE-A + CM/SM	5–10%
HAT + SM + HVA	50%

* Patients suffering from the combined MCAS type are at high risk to develop life-threatening anaphylaxis despite prophylactic anti-mediator therapy. Abbreviations: MCAS, mast cell activation syndrome; IgE, immunoglobulin E; HAT, hereditary alpha tryptasemia; HVA, hymenoptera venom allergy.

Table S3. Variants of MCAS and diagnostic features (criteria).

Variant of MCAS	Main Diagnostic Features
Primary MCAS (= Clonal MCAS) *	the *KIT* D816V mutation is detected and mast cells the *KIT* D816V mutation is detected and mast cells a) with confirmed mastocytosis (CM or SM) ** b) with only two minor SM criteria **
Secondary MCAS	an IgE-mediated allergy, another hypersensitivity reaction or another immunologic disease that can induce MCA and thus MCAS, is diagnosed, but no neoplastic MC or *KIT* D816V is found ***
Idiopathic MCAS	criteria to diagnose MCAS are met, but no related reactive disease, no IgE-dependent allergy, and no neoplastic/clonal MC are found

* The terms clonal MCAS, monoclonal MCAS (=MMAS) can be used synonymously with the term primary MCAS. ** Most of the patients suffer from CM or SM. However, in some cases, only two minor SM criteria are detected and criteria for SM and CM are not fulfilled. *** No *KIT* mutation at codon 816 is detected, and flow cytometry (if performed) will not detect a clonal population of CD25-positive MC. Abbreviations: MC, mast cells; MCA, MC activation; CM, cutaneous mastocytosis; SM, systemic mastocytosis.
